# Lung Nodule Evaluation Using Robotic-Assisted Bronchoscopy at a Veteran’s Affairs Hospital

**DOI:** 10.3390/jcm10163671

**Published:** 2021-08-19

**Authors:** Chigozirim N. Ekeke, Matthew Vercauteren, Smiljana Istvaniczdravkovic, Roy Semaan, Rajeev Dhupar

**Affiliations:** 1 Department of Cardiothoracic Surgery, Division of Thoracic and Foregut Surgery, University of Pittsburgh School of Medicine, Pittsburgh, PA 15232, USA; ekekecn@upmc.edu; 2 VA Pittsburgh Healthcare System, Surgical Services Division, Pittsburgh, PA 15240, USA; matthew.vercauteren@va.gov (M.V.); Smiljana.istvaniczdravkovic@va.gov (S.I.); semaanrw2@upmc.edu (R.S.); 3 Department of Medicine, Division of Pulmonary, Allergy, and Critical Care Medicine, University of Pittsburgh School of Medicine, Pittsburgh, PA 15232, USA

**Keywords:** robotic bronchoscopy, lung cancer, robotic surgery, navigational bronchoscopy

## Abstract

The incidence of lung nodules has increased with improved diagnostic imaging and screening protocols. Despite improvements for diagnosing pulmonary nodules with technologies such as electromagnetic navigational bronchoscopy (ENB), several limitations still exist including adequate visualization, localization, and diagnostic yield. Robotic-assisted bronchoscopy with ENB has been introduced as a method to overcome these shortcomings. We describe our initial experience in evaluating lung nodules with robotic assisted bronchoscopy. We retrospectively reviewed data on the first 25 patients that underwent robotic-assisted bronchoscopy and biopsy. We analyzed success with localization, diagnostic yield, and post procedural morbidity. Diagnostic yield was 96% (24/25) with no periprocedural morbidity. The majority of nodules were malignant or atypical (76%) and were located in the right upper lobe. Diameter ranged between 0.8–6.9 cm (median size 1–2 cm). Seventy-five percent of patients underwent subsequent treatment for cancer based on these results, with 25% having continued surveillance. Robotic assisted bronchoscopy is safe and accurate. Studies with larger numbers will allow better understanding of the diagnostic yield and clinical utility of this approach in comparison to other diagnostic tools for lung nodules.

## 1. Introduction

Over one million lung nodules are detected every year in the United States [1]. This incidence is increasing, as is the need to improve diagnostic yield for lung nodules. Improvements in technology have allowed an evolution in approaches from transbronchial biopsy to computed topography (CT) guided biopsy, endobronchial ultrasound with combined transbronchial biopsy, and more recently electromagnetic navigational bronchoscopy (ENB). Despite these advances, several limitations to a high diagnostic yield remain, such as the ability to visualize a target or the technical challenges of a target location. For example, fiberoptic bronchoscopy with transbronchial biopsy is limited to larger, central lesions, and has a modest diagnostic yield (18–60%) [2]. Percutaneous CT guided biopsy provides the advantage of a 70–90% yield when targeting peripheral lesions, but this is highly dependent on location and size [3,4]. There are also adverse effects such as pneumothorax requiring intervention (1–15%), pulmonary hemorrhages (18%) and hemoptysis (4.1%) [3,5,6]. While the adverse effects are less with ENB (pneumothorax 3.2%, pulmonary hemorrhage 2.3%), and the overall diagnostic yield ranges from 59–77%, it is a technically challenging procedure without the ability to directly visualize the target [7,8]. 

Recently, robotic-assisted bronchoscopy coupled with ENB was introduced with the intention to not only improve diagnostic yield, but also navigation and visualization [9]. Robotic bronchoscopy coupled with ENB allows surgeons and interventional pulmonologists to directly visualize central and peripheral lung nodules, in contrast to its technological predecessors. Recent papers have shown robotic bronchoscopy with ENB to be feasible with minimal complications [9,10]. Few studies review clinical outcomes, and most are limited to a small cohort [10,11,12]. The following describes our initial experience in evaluating lung nodules with robotic bronchoscopy.

## 2. Materials and Methods

We conducted a review of the first 25 patients that underwent robotic bronchoscopy with ENB at the Veteran’s Affairs Pittsburgh Healthcare System (VAPHS). Due to the makeup of the veteran population, 100% of patients in our study are male. This quality initiative for a new technology was approved by the VAPHS IRB as not being research. Data was reviewed from CPRS and Auris’ robotic bronchoscopy platform with permission from the company (Monarch^TM^ platform by Auris Health, Inc., New Brunswick, NJ, USA) between August 2020 and February 2021. Characteristics and descriptions of lung nodules were obtained from the CT scan reports preceding the robotic bronchoscopy procedure. The presence of a bronchus sign was determined after the subjects’ scans were analyzed with the Monarch Planning System and a computer-generated path was created in parallel with the target anatomy. Central, segmental, and subsegmental nodules were classified based on their relative locations to the main lobar, segmental, and subsegmental bronchi. Central lesions that were accessible by conventional bronchoscopy were not included, as they did not require use of this platform. We also collected the following data: demographic information, nodule location, final biopsy pathology (Figures S1 and S2), presence of post-procedural morbidity, number of biopsies, and ultimate treatment. When the pathology was benign and not hamartomatous, the results were lung parenchyma with inflammatory changes.

### 2.1. Pre-Procedural Assessment

The patient’s most recent CT scan of the chest is uploaded into the Monarch Planning System. A member of the procedural team identifies the lung nodule(s) of interest to construct a three-dimensional model of the nodule and bronchial anatomy. A positive bronchus sign occurs if the computer software generates a path that leads directly to a nodule. A negative bronchus sign may require repeat CT scan (with IV contrast) using the following parameters recommended by the company: 0.8 mm interval with slice thickness of 1 mm. If a negative bronchus sign remains, a path to the target anatomy can be created manually by placing “way points” available within the software, starting from the nodule to the end of the previously completed computer generated path. Typically, these way points follow closely to a blood vessel or suspected airway that was not automatically created by the Monarch Planning System.

### 2.2. Patient Positioning and Anesthesia Considerations

The patient is supine on the procedure table. After endotracheal intubation with an 8.0 mm (or larger) tube, the bed is positioned to allow the operating team easy access to the airway. Fiberoptic bronchoscopy is performed to position the distal end of the endotracheal tube (ETT) 4 cm from the main carina. A survey of the endobronchial anatomy is performed and mucous is cleared. Bronchodilators and positive end expiratory pressure can be set between 6–8 cm H_2_O to maximally open airways. Figures S3–S7 illustrate the positioning and set-up.

### 2.3. Robotic-Assisted Bronchoscopic Procedure

Electromagnetic pads are connected from the robotic cart to the patient, 2 cm below the sternal notch and at the anterior axillary line in the 8th intercostal space bilaterally. Ideal pad placement is confirmed on the robotic platform monitor. The field magnet is attached to the procedure table on the ipsilateral side of the target lesion at the level of the costal margin and is pointed toward the contralateral shoulder.

The robotic ETT holder is then attached to the procedure table at the level of the tragus on the ipsilateral side of the target. The ETT is cut at the level of the tip of the patient’s nose and a new ETT bronchoscope adaptor is placed to ensure complete pneumostasis throughout the procedure. The ETT is then secured to the robotic ETT holder, confirming a straight and upright orientation (perpendicular to the patient) with care to not exert any undue stress on the patient’s mouth and face. The robot is positioned at the head of the bed and is locked in place. The arms are deployed and the arm nearest to the patient is aligned manually at the ETT holder using the alignment markings. The suction/irrigation tubing and robotic camera apparatus are attached to the arms and advanced into the ETT to visualize the carina.

**Figure 1:**
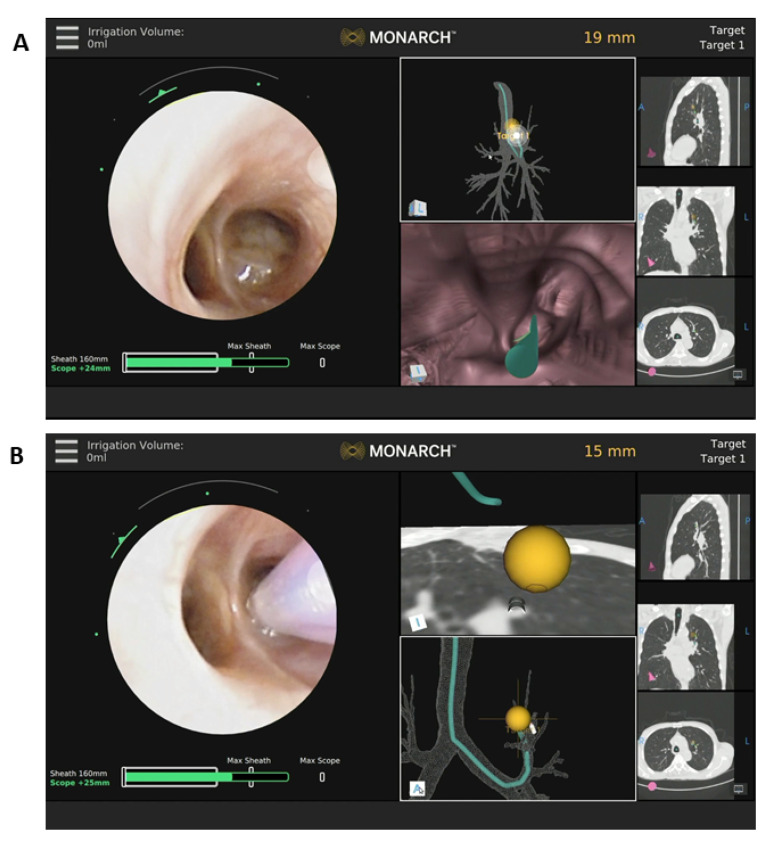
Example of images from the monitor during a robotic bronchoscopy procedure. (**A**) The left part of the screen shows the bronchoscopic image of the left upper lobe sub-segmental bronchus with endobronchial tumor. The middle images show the generated pathway to the left upper lobe nodule and the right images show the accommodating sagittal, coronal and axial views of the tumor in the left upper lobe. (**B**) Needle biopsy of the tumor shown in A.

Camera orientation is adjusted prior to registration of the bronchoscopic images with the navigation software. Upon registration completion, the operator can begin the procedure. Set up time is approximately 5 minutes after 10 cases have been conducted by the same team. The proceduralist drives the scope to the target lesion(s) with the hand-held controller and biopsies can be performed with a needle or forceps (Figure 1).

## 3. Results

Twenty-five patients underwent robotic bronchoscopy for evaluation of a lung nodule. Patient and nodule characteristics are summarized in (Table 1). All patients were male, with a median age of 71 and BMI of 26. The nodule locations varied with right upper lobe as the most common site, followed by the left upper lobe. Lung nodule size ranged from 0.8–6.9 cm, 54% being under 2 cm in diameter.

A median of four biopsies were performed with a needle and/or forceps. Adequate sampling with an actionable diagnosis was achieved for 96% of the patients (Table 2). Final pathology revealed malignancy in 15 patients (60%) and highly suspicious/atypical cells in 4 patients (16%). Of those four patients, one patient underwent lung resection and on final pathology had non-small cell lung cancer (Table 3). Benign nodules were identified in five patients (20%), with evidence of hamartoma in two out of the five cases. Four patients (16%) continued surveillance due to benign findings.

During the study period there were no reported post-procedure admissions, morbidities, or mortalities secondary to the procedure.

## 4. Discussion

With an increasing of incidence of lung nodules, several bronchoscopic techniques have evolved to improve diagnostic yield while limiting morbidity. Indeed, current approaches are largely based on patient characteristics, nodule location and anatomy. Despite the use of CT guided biopsy, endobronchial ultrasound, and ENB, 25–30% of procedures are nondiagnostic [10]. The associated morbidities (pneumothorax, pulmonary hemorrhage, need for further interventions) remain a challenge and are would potentially be avoided with improved navigation and visualization. Robotic bronchoscopy with ENB is a safe and effective way to obtain lung nodule biopsies from both central and peripheral lung nodules. ENB without robotic navigation has been used successfully to localize pulmonary nodules for biopsy and resection, though such good results are not wide-spread [13].

Our series demonstrates no evidence of morbidity or need for additional interventions following the use of this robotic platform, which echoes what has been demonstrated by others [9,10,11]. Chaddha and colleagues’ multicenter experience with a robotic platform reviewed 160 patients, showing most of the lesions were malignant and were located in the right upper lobe, much like ours. The diagnostic yield in that study ranged between 69–77% and morbidity (pneumothorax, hemoptysis) occurred in less than 4% of the cases [11]. Rojas et. al. assessed 15 patients following robotic bronchoscopy and found no post procedural morbidity [9].

There are limitations to our data, such as selection bias and the inability to compare this group of patients to a matched cohort that underwent alternative diagnostic procedures. However, in this group we did not select patients for other diagnostic procedures due to location or lack of a “bronchus sign”; these patients were consecutive. Ideally, a prospective, randomized trial or a robust comparison of diagnostic yield among patients who underwent robotic bronchoscopy versus other procedures (e.g. transbronchial biopsy, CT guided biopsy, or legacy ENB) will be performed. Despite these limitations, our study of the quality of robotic-assisted ENB for the biopsy of lung nodules is encouraging. Specifically, the advantage of visualization in the airways precludes the need for a bronchus sign or complete path as the proceduralist can still navigate towards a target by seeing the airways.

In conclusion, robotic-assisted bronchoscopy can successfully obtain sufficient samples from lung nodules to aid treatment decisions. We believe this approach can be embraced by thoracic surgeons and interventional pulmonologists, as we see a rising incidence of lung nodules and in the near future expect therapeutic interventions will be available for use in conjunction with such platforms [14].

## Figures and Tables

**Table 1:** Patient and target characteristics (*n* = 25).

Patients	*n*	%
Gender (male)	25	100
Age (median, years)	71	-
BMI (median)	26	-
Tobacco use	23	92
Targets		
<1 cm	2	8
1–2 cm	11	44
2.1–3 cm	6	24
>3.1 cm	6	24
Right upper lobe	9	36
Right middle lobe	2	8
Right lower lobe	4	16
Left upper lobe	7	28
left lower lobe	3	12
Lobar bronchus	3	12
Segmental bronchus	6	24
Subsegmental bronchus	16	64
Bronchus sign		
Yes	21	84
No	4	16

**Table 2:** Pathologic findings.

Pathology	*n*	%
Malignant	15	60
Atypical/Suspicious	4	16
Benign	5	20
Other	1	4

**Table 3:** Treatments based on biopsy results.

Treatments	*n*	%
Surgical resection and adjuvant therapy (if needed)	8	32
Stereotactic radiation	7	24
Chemotherapy ± radiation therapy	5	20
Surveillance imaging	5	25

## Data Availability

All data are available upon request from the corresponding author (RD).

## Supplementary Materials

The following are available online at https://www.mdpi.com/article/10.3390/jcm10163671/s1. Figure S1: Representative FNA by robotic bronchoscopy from a nodule with maligant cells; Figure S2: Represetnative biopsy by robotic bronchoscopy from a cancer. Figure S3: Auris Monarch Robot. The Robot in the stowed position (A), and with the arms deployed from the front (B) and side (C) views.; Figure S4: Positioning and Anesthesia Considerations. The endotracheal tube holder is attached to the bed at the level of the tragus and cut at the level of the nose. A bronchoscope adapter is subsequently attached.; Figure S5: Electromagnetic pad application. The field magnet is attached to the table on the ipsilateral side of the target anatomy and the electromagnetic pads are placed on the patient’s chest within the electromagnetic field (as determined by the Monarch software).; Figure S6: Securing the endotracheal tube. The ventilator tubing is secured onto the endotracheal tube holder to avoid access pressure and air leaks at the junction between endoscope entry into the endotracheal tube (A and B).; Figure S7: Securing the bronchoscope. The robotic bronchoscope is inserted into the sheath and attached to the bedside cart (A). Passage of the bronchoscope encounters little obstruction if the assistant pushes down on the bronchoscope during insertion and initial navigation into the trachea (B).

## Publications/Presentations

Data have not been published and are not submitted for publication elsewhere. They have been presented at the Association of VA Surgeons Meeting in 2021 (virtual).
