# Supplementary figures and images for: Lung Nodule Evaluation Using Robotic-Assisted Bronchoscopy at a Veteran’s Affairs Hospital
Source: J Clin Med. 2021 Aug 19;10(16):3671. doi: 10.3390/jcm10163671 (PMC8397153; doi:10.3390/jcm10163671)

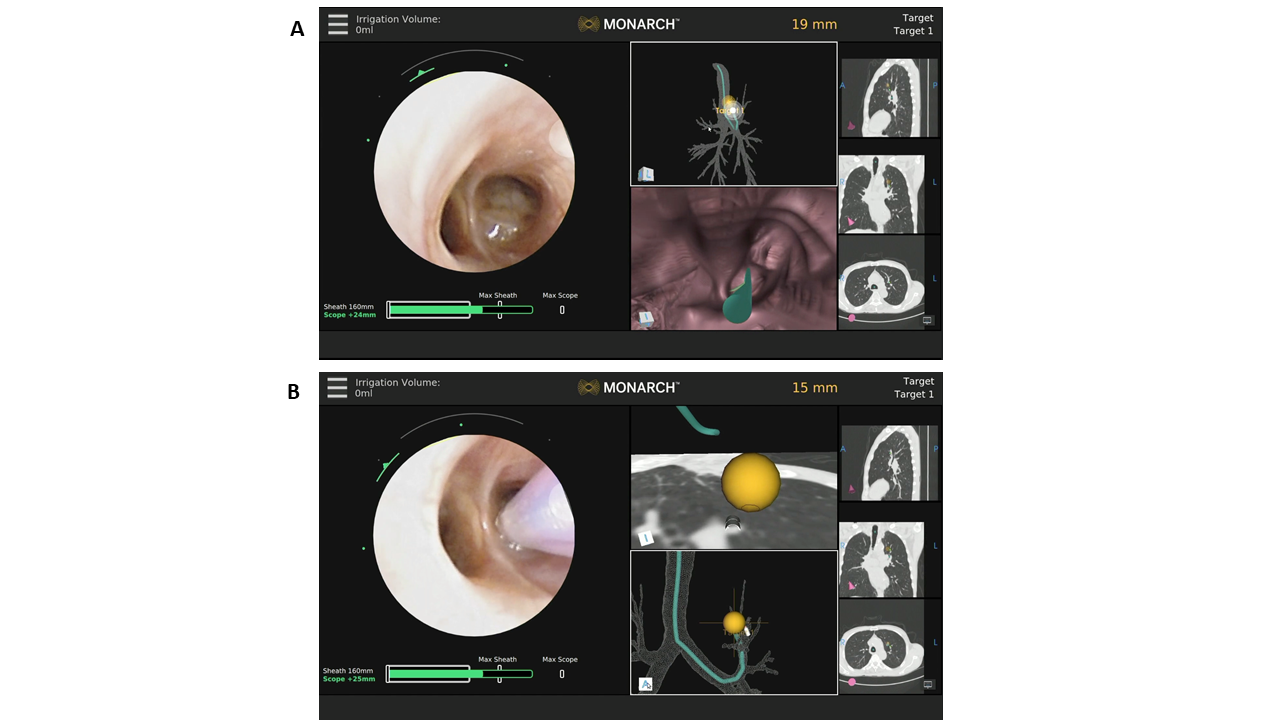

Supplement: Supplementary file 1 [file jcm-10-03671-s001.zip › Figure 1.TIF]

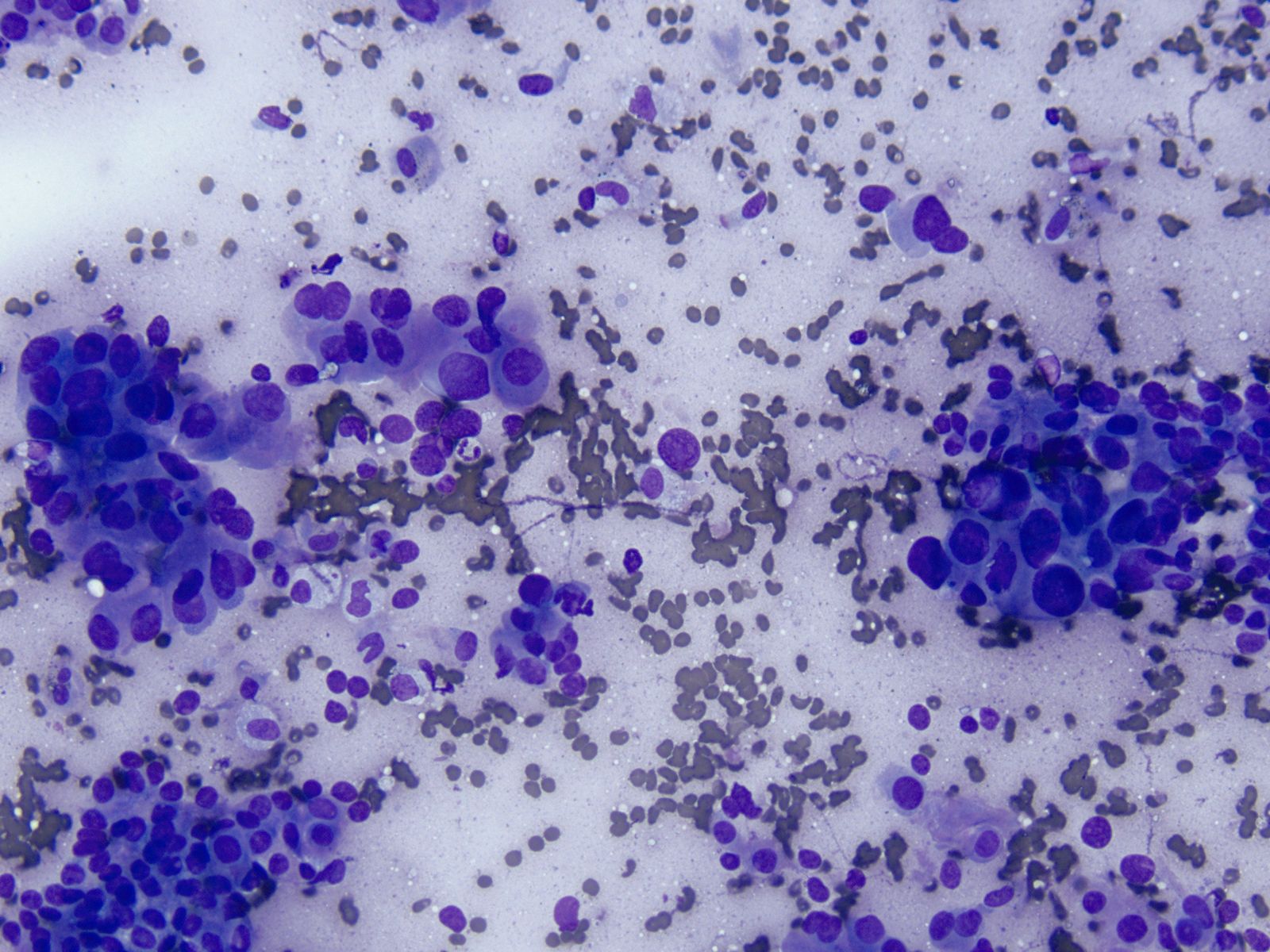

Supplement: Supplementary file 1 [file jcm-10-03671-s001.zip › Figure S1.jpg]

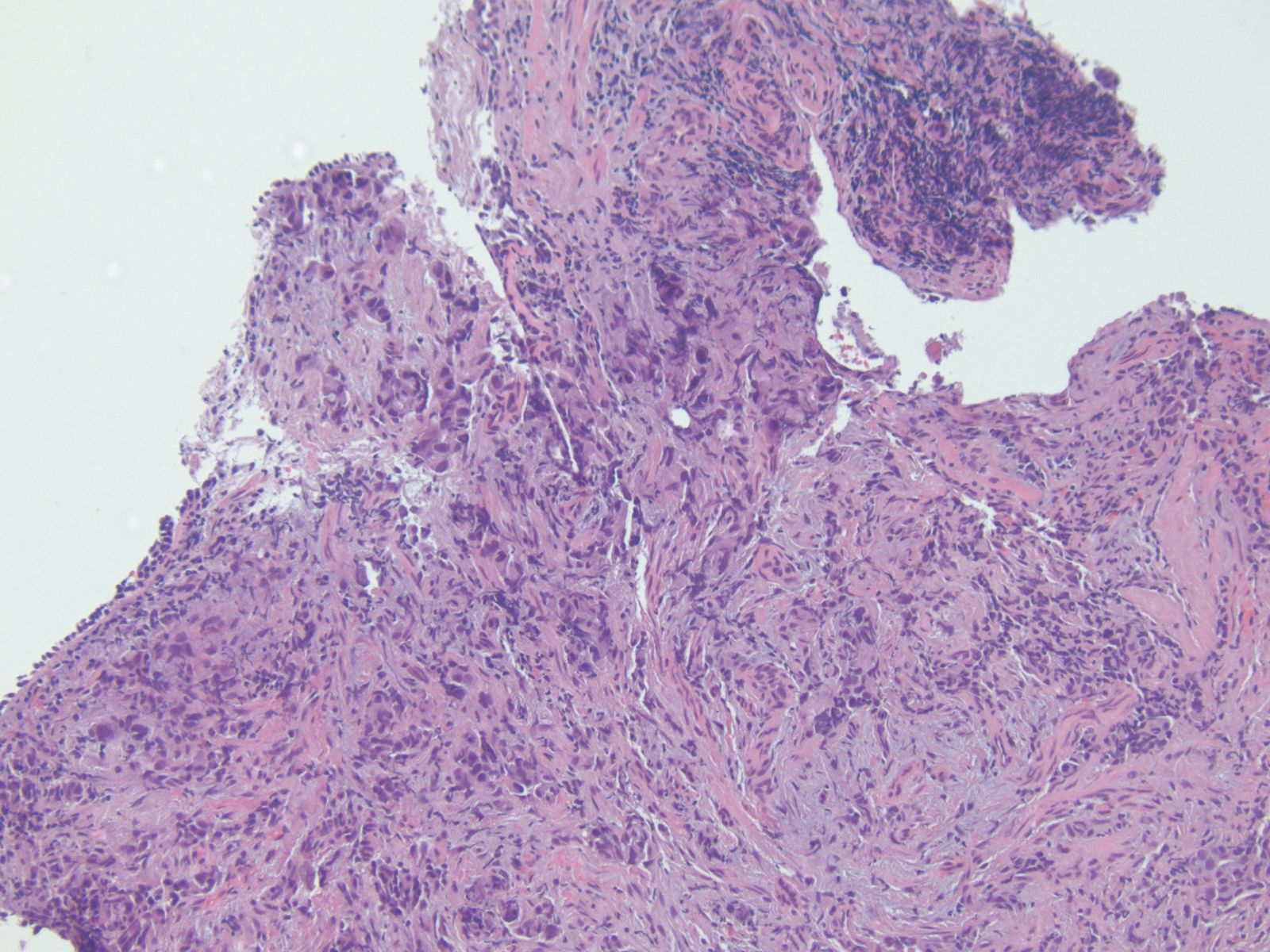

Supplement: Supplementary file 1 [file jcm-10-03671-s001.zip › Figure S2.jpg]

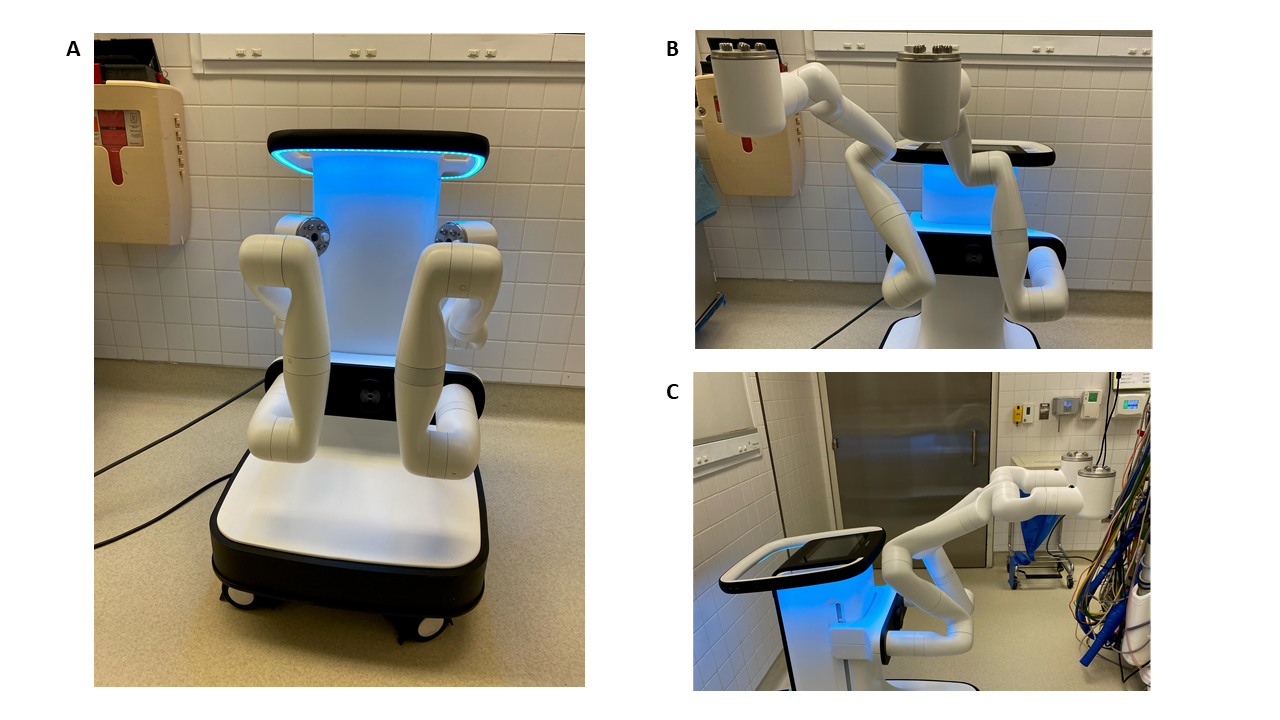

Supplement: Supplementary file 1 [file jcm-10-03671-s001.zip › Figure S3.TIF]

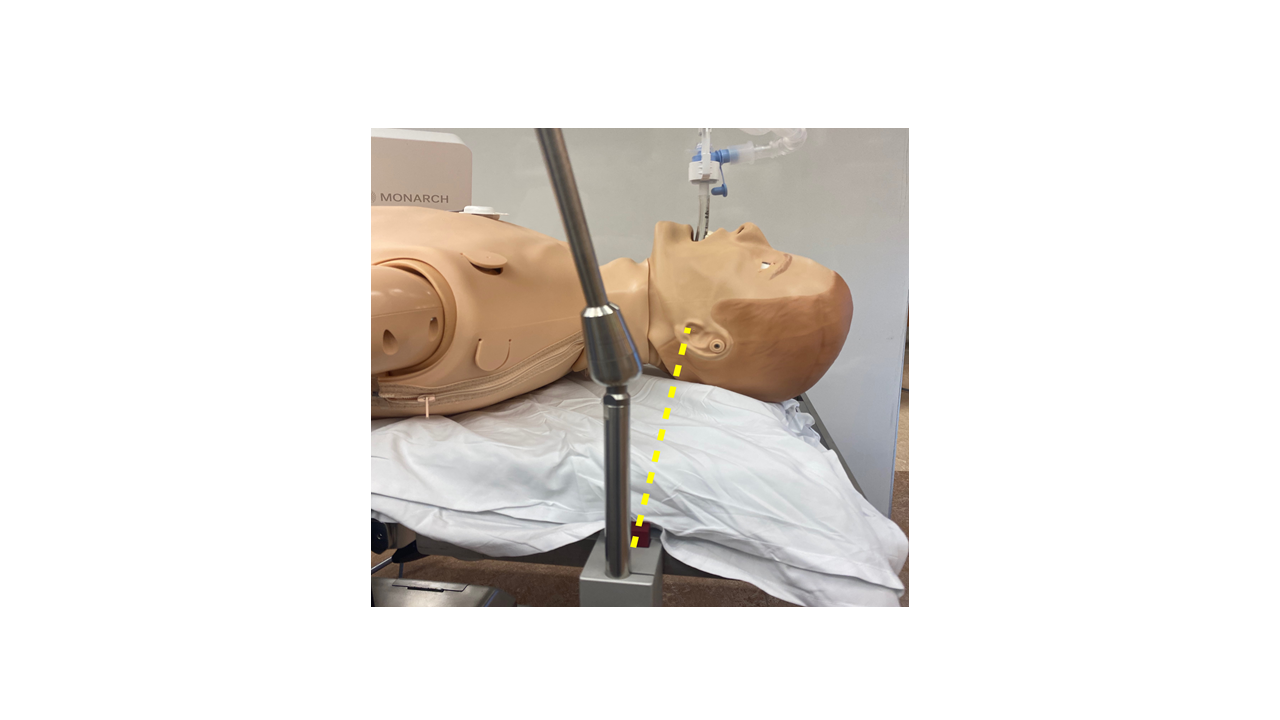

Supplement: Supplementary file 1 [file jcm-10-03671-s001.zip › Figure S4.TIF]

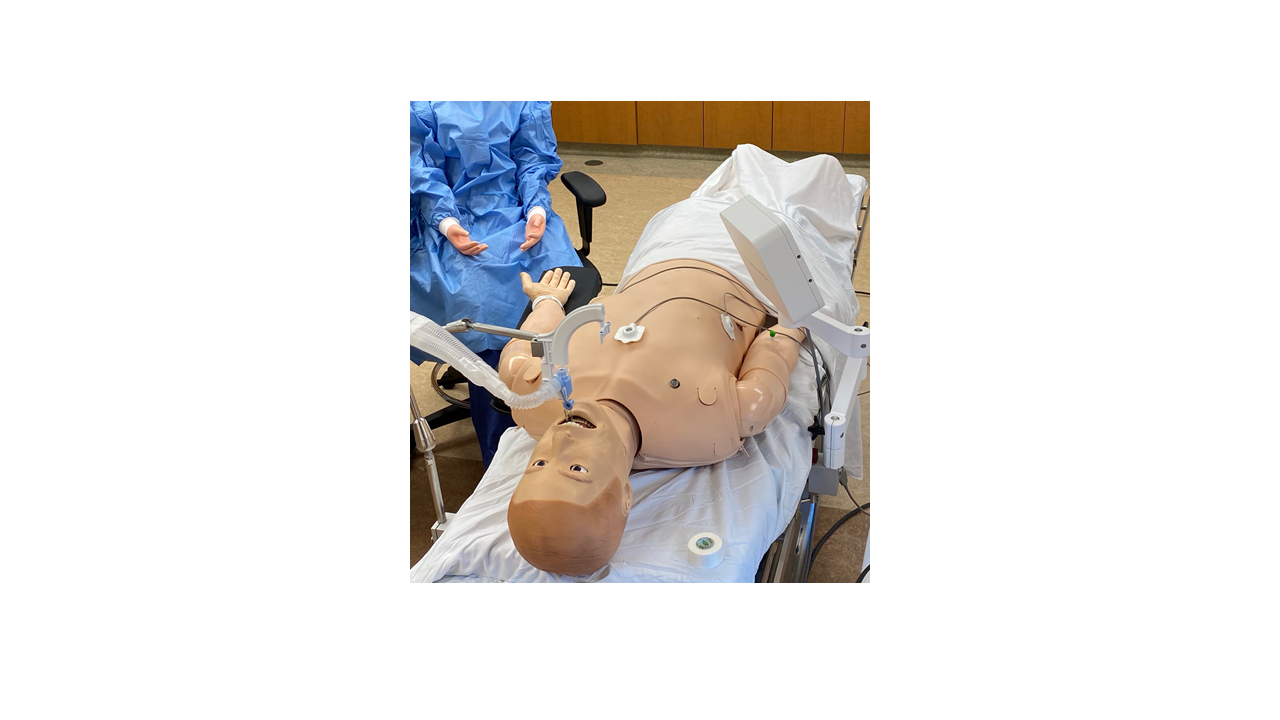

Supplement: Supplementary file 1 [file jcm-10-03671-s001.zip › Figure S5.TIF]

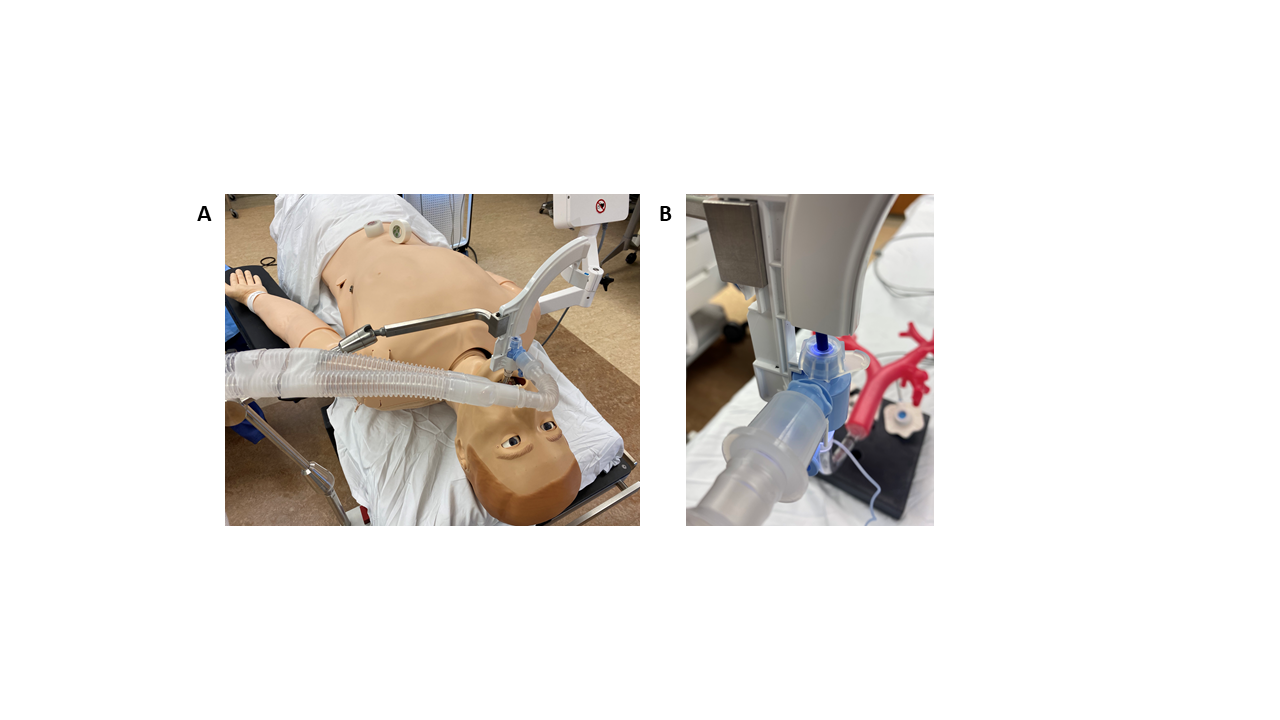

Supplement: Supplementary file 1 [file jcm-10-03671-s001.zip › Figure S6.TIF]

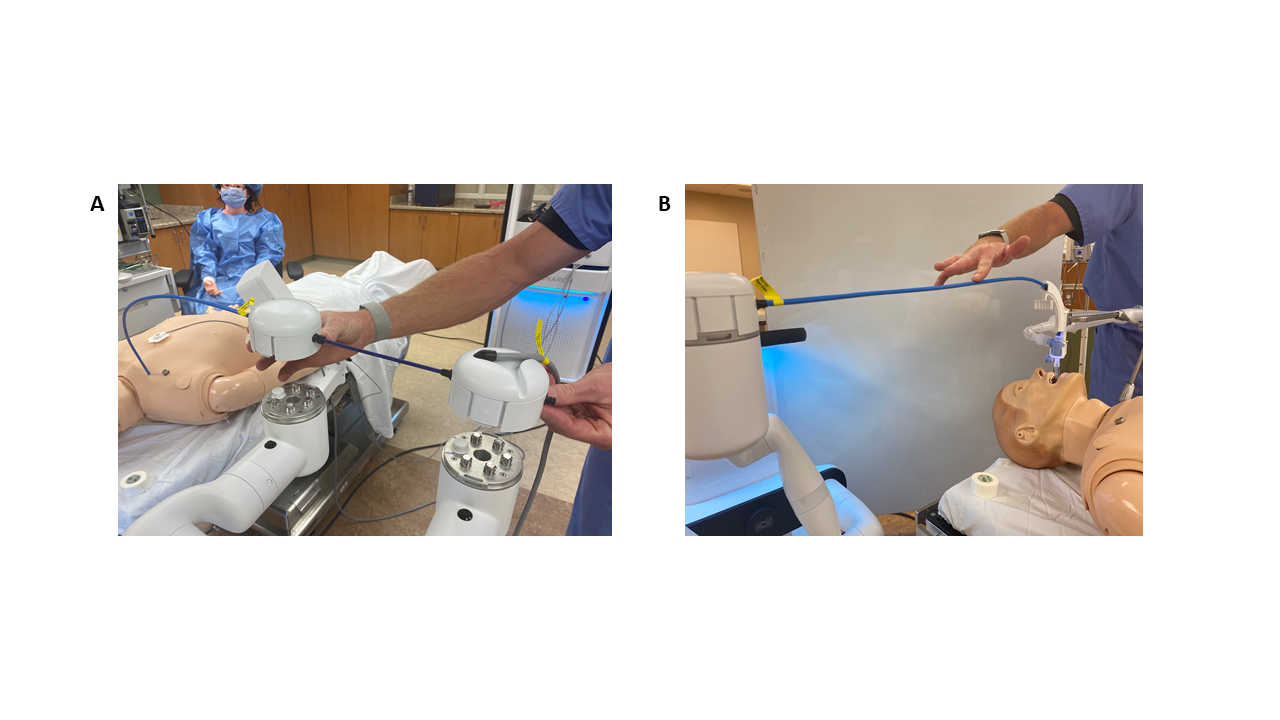

Supplement: Supplementary file 1 [file jcm-10-03671-s001.zip › Figure S7.TIF]
